# The Role of T Cells in Herpes Stromal Keratitis

**DOI:** 10.3389/fimmu.2019.00512

**Published:** 2019-03-19

**Authors:** Naveen K. Rajasagi, Barry T. Rouse

**Affiliations:** Biomedical and Diagnostic Sciences, College of Veterinary Medicine, The University of Tennessee, Knoxville, TN, United States

**Keywords:** herpes stromal keratitis, CD4 T cells, metabolism, regulatory T cells, plasticity

## Abstract

The blinding inflammatory lesion stromal keratitis (SK), which occurs in some patients in response to ocular herpes simplex virus (HSV) infection, represents mainly an immune cell mediated inflammatory response to the virus infection. The principal orchestrators of the immunopathological lesions are T cells although additional events participate that include the extent of recruitment of non-lymphoid cells, the extent of neoangiogenesis, and the extent of damage to nerve function. This review focuses on evidence that the balance of the functional subsets of T cells has a major impact on lesion severity and duration. Accordingly, if proinflammatory Th1 and Th17 CD4 T cells, and perhaps in some cases CD8 T cells, predominate lesions occur earlier and are more severe. Lesions are diminished when cells with regulatory function predominate. Moreover, when regulatory cells acquire the property to produce Amphiregulin this may facilitate lesion resolution. An objective to controlling lesions is to learn how to manipulate the balance of T cells to favor the representation and function of regulatory T cells and their products over proinflammatory cells. In this review we emphasize how exploiting the differential metabolic requirements of immune cells could be a valuable approach to control SK.

## Background

Herpes simplex virus (HSV) type 1 is a major human pathogen worldwide. It is estimated that around 67% of people worldwide (under age 50) are infected with HSV-1 (1). HSV-1 establishes a lifelong, latent infection for which no effective vaccine is currently available (2). Primary infection with HSV-1 is usually mild or subclinical and most individuals remain asymptomatic (3). However, HSV-1 infection can cause several complications in humans. Among these, corneal infection can lead to blinding immunopathological lesions in the eye referred to as herpes stromal keratitis (SK) (4, 5). Epidemiology studies outside of the United States have estimated incidence rates of HSV eye disease range from ~4 to 13 new cases per 100,000 per year. A previous study from Rochester, Minnesota, estimated an incidence of 8.4 new cases per 100,000 and 20.7 total episodes per 100,000 people per year. Extrapolating these data to the US population census in 2000, the study predicted an estimated incidence of ~24,000 new cases and 58,000 total episodes per year (6). Moreover, a study published in 2014, estimated an incidence of 6.8 new cases/100,000 in Northern California (7). Thus, herpes keratitis represents a clinically relevant syndrome and the SK form is a frequent cause of vision damage.

Primary ocular infection most likely occurs by the direct infection of the eye with HSV-1. Upon infection, the virus replicates in the corneal epithelial cells and can causes epithelial lesions. These primary lesions can last up to 2 weeks and usually resolve with minimal damage and the virus is efficiently cleared by the immune system (8). However, one of the consequences of HSV ocular infection is the establishment of latency in the trigeminal ganglia (TG) (9). Some of the HSV virions can enter the sensory nerve endings which innervate the infected cells and traffic via retrograde transport mechanisms to the sensory ganglia where the virus can persist in a latent stage (10). Sometimes the latent virus reactivates by disturbances caused by environmental or physiological stress and the reactivated HSV replicates in the TG. The virus can then travel by anterograde axonal transport to the peripheral tissues and cause recurrent lesions either in the corneal or orofacial tissues often resulting in clinical consequences (11). In humans, recurrent virus infections of the cornea are usually confined to the epithelial layer, but in some individuals such frequent recurrent infections could affect the deeper corneal stroma leading to an immunopathological disease referred to as herpes stromal keratitis (SK). This chronic inflammatory response in the corneal stroma is mediated by both innate and adaptive immune cells in response to virus infection and can lead to progressive corneal scarring and vision loss. The local corneal epithelial lesions and virus infections are usually treated using antivirals such as acyclovir, but SK lesions are often treated with a combination of an antiviral and a corticosteroid (12).

Most of our current understanding of the pathogenesis of SK in humans comes from studies done animal models (5, 13). HSV-1 corneal infection in mice is the most widely used animal model to study SK as it offers several advantages and the inflammatory lesions in the corneal stroma mimic SK lesions observed in humans (14). However, one limitation of the mice model is that it is mainly a primary infection model, but not a reactivation model of disease as mostly occurs in humans. The immune response to HSV-1 ocular infection occurs in a bi-phasic manner and involves both innate and adaptive components of the immune system (8). During the pre-clinical or acute phase, the first wave of immune cells mainly consisting of neutrophils, natural killer cells, and macrophages enter into the corneal stroma and help to clear the replicating virus (5). In the later clinical or chronic phase of the disease, CD4 T cells start to appear in the cornea around day 6–7 post-infection, a stage when virus is usually already cleared from the cornea (8). The CD4 T cells are considered to be the primary orchestrators of SK lesions as they facilitate the influx of the second wave of neutrophils (15). The massive cellular infiltration especially neutrophils coupled with the inflammatory mediators secreted by the immune cells are primarily responsible for the swelling and destruction of the cornea (16, 17).

## Role of Th1, Th17, and CD8 T Cells in SK Lesions

Stromal keratitis (SK) is an immunopathological disease orchestrated by T cells (14). This view is supported by findings which show that mice depleted of T cells are less susceptible to HSV-1 induced corneal stromal disease. In both humans and mice, there is a predominance of CD4 T cells in the ocular tissues during SK and their functional activities are often associated with the tissue damage in the corneal stroma. In mice, CD4 T cells appear in the corneas around day 6 post-ocular infection with HSV-1 and their numbers continue to increase during the latter stage of SK development. Among the CD4 T cell population, there is a preferential accumulation of CD4 T helper (Th1) subset in the eye (18). Th1 cells express the transcription factor, T-bet, and produce various immune-modulatory mediators which play a role in SK lesion expression. The Th1 cells secrete the cytokines IFN-γ and IL-2 which are capable of inducing corneal inflammation and neovascularization (19, 20). In addition, these cytokines also modulate chemokine factors, and in doing so could facilitate the massive influx of neutrophils and macrophages into the cornea during the latter phase of SK development (21, 22). Another CD4 subset which gained recent prominence in inflammation and autoimmunity are the Th17 cells (23). These cells express the transcription factor ROR-γt and produce cytokines such as IL-17, IL-21, and IL-22. They preferentially produce IL-17 which is a potent inducer of additional pro-inflammatory cytokines, chemokines, and metalloproteinases (24, 25). Th17 cells accumulate in the HSV infected cornea during the later stages of SK pathogenesis and help sustain and expand the disease (26, 27). Moreover, HSV-1 ocular infection of IL-17R knock-out mice or neutralization of IL-17 using monoclonal antibodies delayed disease progression and reduced the severity of HSK (26). Importantly, IL-17 was expressed in corneas of patients with SK (28). In addition, the human corneal fibroblasts constitutively express the IL-17R. The data from these studies suggest that IL-17 strongly induces the production of key inflammatory mediators such as IL-6, IL-8, and matrix mettalloproteinase-1 in the human corneal fibroblast cultures (28). Thus, Th17 cells through the production of IL-17 modulate the levels of chemotactic factors such as CXCL-1 and IL-8 and influence the migration of neutrophils into the inflamed corneal tissues (26).

Although, CD4 T cells are considered to be the chief perpetuators of SK, the data presented in some experimental models implicate CD8 T cells in the pathogenesis of SK. The outcome depends to a large extent on the virus strain used for the studies. Some studies found that ocular infection of mice with the HSV-1 RE strain mainly induces SK mediated by CD4 T cells, whereas infection of the same stain of mice with HSV-1 KOS show SK which is dependent on CD8 T cells (29). In mice infected with a recombinant stain of HSV-1 (HSV-gK), the corneal scaring and the corneal disease were mainly mediated by CD8 T cells (30, 31). Results from these studies suggest that gK strongly induces CD8 T cell responses leading to exacerbation of SK lesions. Of note, the recombinant HSV-gK strain used in these studies contains three copies of glycoprotein K (gk) (a protein essential for virus replication) compared to one copy in the wild type HSV-1 McKrae strain (30). The HSV-1 mutant strains which lack gK were found to be defective in infectivity and failed to establish latency in the neurons in mouse models which suggests that gK expression is crucial for virus replication (32). Thus, the respective roles of different CD4 and CD8 subsets in SK is not clear and remains an unresolved issue. Additionally, some evidence shows that CD8 T cells mainly play more of a protective role (33). Observations in both mice and humans show that HSV-1 specific CD8 T cells are selectively retained in the TG and might help control HSV reactivation (34–36). These tissue resident CD8 T cells appear to use IFN-γ and non-cytolytic mechanisms to block virus reactivation in the TG (37, 38).

## Role of Regulatory T Cells (Treg) in SK Pathogenesis

A beneficial subset of CD4 T cells in SK are regulatory T cells (Treg) (39, 40). Treg express the master transcription factor, Foxp3 which controls their development, and function (41). Treg are either produced as a functionally mature T cell sub population in the thymus (natural Treg) or are induced in the periphery from naive CD4 T cells (induced Treg). Treg mainly function to maintain tolerance to self-antigens and prevent autoimmune diseases (42). They also constrain excessive immune responses to non-self-antigens or infectious agents and help to maintain peripheral tolerance and immune homeostasis (41). Treg use several mechanisms to suppress aberrant immune responses and these include immunomodulatory cytokines (IL-10, TGF-β, IL-35) or contact dependent suppression (granzyme/perforin) (41, 43, 44). In addition, Tregs also exert their function on effector T cells through inhibitory molecules such as CTLA-4. Treg also condition dendritic cells to secrete indoleamine 2,3-dioxygenase, a molecule which suppresses the activation of effector T cells (44).

During microbial infections, a major function of Treg is to control the excessive inflammatory responses to prevent collateral tissue damage and limit injury to the host. In HSV-1 ocular infection, Treg were shown to be crucial to control HSV induced corneal immunopathology. SK lesions were more severe if mice were depleted of Treg before infection using monoclonal antibody treatment, whereas adoptive transfer of *in vitro* converted Treg suppressed HSK severity (45, 46). Furthermore, findings using the depletion of regulatory T cells (DEREG) transgenic mice showed that lesions became more severe even when depletion was begun in the later phases (clinical/chronic phase) of the disease (47). The DEREG mice carry the diphtheria toxin receptor-enhanced green fluorescent protein (DTR-eGFP) transgene under the control of an additional Foxp3 promoter, which facilitates specific depletion of Treg by application of diphtheria toxin at any chosen point of time (48). Thus, measures to expand the representation of Treg by the administration of various reagents have been useful in reducing the severity of SK lesions in the mouse model. One such approach used was galectin-9 which induces apoptosis of pathogenic CD4 Th1 cells and increases the representation of the anti-inflammatory Treg population (49). In addition, a combination treatment using a tumor necrosis factor receptor superfamily member 25 (TNFRSF25) agonist antibody which expands Treg numbers along with galectin-9 was particularly effective in diminishing HSV-1 induced corneal immunopathology (50). Other approaches that were successful in expanding Treg population and reducing SK lesions included the use of IL-2/anti-IL-2 mAb complexes and the fungal metabolite drug, fingolimod hydrochloride (FTY720) (51, 52). In addition, phosphorylated FTY720 also targets sphingosine-1-phosphate receptor and perhaps diminishes inflammation by modulating lymphocyte trafficking (53).

Although increasing the representation of Treg in lesions is a valuable approach to minimize lesion severity, it has become evident that the Treg population is functionally heterogeneous. Accordingly, some functions are more valuable to achieve control than others. For example, our group recently observed that a function of Treg valuable for resolving SK lesions is their ability to produce amphiregulin (AMP) (54). This molecule acts to facilitate tissue repair by binding to the epidermal growth factor receptor expressed mainly on epithelial cells and stem cells and its binding can result in the activation of downstream signaling kinases resulting in growth, proliferation, and migration of cells (55). Treg that produce AMP are relatively infrequent in the early stages of SK, but their representation is most evident in later stages. The change of Treg function to become AMP producers appears to be driven by the cytokines IL-12 and IL-18. In fact, exposure of AMP negative Treg cells *in vitro* to these cytokines can induce them to become AMP producers. In addition, if animals were treated *in vivo* with a plasmid which expresses IL-18, this led to the reduced expression of SK lesions, an effect that correlated with a higher frequency of Treg that were AMP producers (54). Finding practical approaches to induce cells in SK to become AMP producers could represent a useful approach to therapy, an issue that merits further investigation.

## Plasticity of Regulatory T Cell Populations

Some recent observations suggest that Treg might become unstable in certain highly inflammatory environments and lose their regulatory activity (56). Under such conditions, Treg that downregulate Foxp3 expression might even take up an effector phenotype and start producing pro-inflammatory cytokines such as IFN-γ and IL-17 Treg, a phenomenon commonly referred to as plasticity (57–59). In recent times, plasticity in T cells has been a matter of debate as it has biological implications especially in therapeutic regimens which use Treg (60, 61). Factors which influence Treg stability are as yet not clear and remains an active area of research. Although multiple mechanism might be involved in the stability and plasticity of Treg, most evidence indicates that Treg stability and Foxp3 expression is controlled by epigenetic mechanisms, namely DNA methylation in the non-coding region (CNS2) of the Foxp3 gene locus, also known as Treg-specific demethylation region (TSDR) (62). Any changes or modifications in the DNA methylation status in the TSDR region tend to have an effect on Foxp3 expression and stability of Treg populations (63). Most Treg populations are generally resistant to destabilization and reprogramming and maintain their transcriptional expression of regulatory genes and functional phenotype (61). Some of the Tregs generated *in vitro* or *in vivo* which have incomplete demethylation status in the cytosine-phospho-guanine (CpG) sites in the TSDR region are more prone to instability when exposed to cytokine milieu containing IL-6, IL-12, IL-21, or IL-23 (57, 64). The Bluestone group, using Foxp3-Cre reporter mice in an Experimental autoimmune encephalomyelitis (EAE) model observed that some of the Treg cells downregulated Foxp3 expression and these were referred to as exFoxp3 cells (59). Such exFoxp3 cells isolated from the CNS at the peak of the response produced IFN- γ when stimulated with cognate antigen (59). Our group using fate mapping mice showed that Treg plasticity can occur in HSV-1-induced inflammatory environment and such Treg may contribute to SK lesion severity by secreting the proinflammatory cytokine IFN-γ (65). In particular, Treg cells showing low expression of the IL-2R (CD25) could exhibit instability, in part due to the exposure to the pro-inflammatory cytokine IL-12 in the cornea (65). In such circumstances, drugs such as azacytidine, retinoic acid, and vitamin C which maintain demethylation of the TSDR region of Foxp3, can be helpful in promoting the stability and improving the functionality of Treg especially under chronic inflammatory conditions (65). In fact, in a recent study, Treg generated *in vitro* in the presence of Azacytidine expressed a fully demethylated TSDR and these cells displayed enhanced suppressive activity (66). Moreover, administration of 5-Azacytidine reduced the incidence of SK lesions in mice infected ocularly with HSV-1 (66).

## Manipulating Metabolism to Constrain SK Lesions

In the previous section, we have argued that the clinical expression of SK is affected by the representation of different participants in lesions. When the T cell participants were dominated by Treg, lesions will be less severe and may even resolve. Hence, a potentially valuable approach to therapy is to use maneuvers that can shift the balance of events away from dominance by proinflammatory components. This therapeutic challenge is also faced by those working with other in other chronic inflammatory diseases, especially autoimmune diseases (AID). In the AID field, some are considering using approaches such as adoptive cell transfer to enrich the population of Treg (67). However, such an approach, which is most effective when the Treg are antigen specific, would likely fail to adequately gain access to the eye. Other approaches include administering reagents that expand the Treg population as we discussed previously. A potentially more useful therapeutic option would be to exploit the accumulating knowledge that cells involved in immune function may differ in the major metabolic pathways they use to provide them with energy and other events that maintain of their various functions (68, 69). For example, proinflammatory and Treg cells use different pathways to provide energy with the former mainly use extracellular glucose and Treg rely on fatty acid oxidation (68). Rathmell's group reported that effector T cells (both CD4 and CD8) express high levels of the glucose transporter Glut1 and utilize the mammalian target of rapamycin (mTOR) pathway to increase glycolysis to support their function (70). In contrast, Treg primarily use AMP-activated protein kinase and rely upon lipid oxidation for their energy. The activated AMPK pathway in Treg acts to inhibit mTOR by suppressing mTOR signaling and promotes mitochondrial oxidative metabolism rather than glycolysis and is considered to be anti-inflammatory (70). In our own studies, we have begun to exploit the differences by which proinflammatory and Treg cells derive their energy needs. We have shown that if glucose utilization is inhibited, as can be achieved by the use of 2 deoxy glucose administration from the initial time of lesion development, that lesions are significantly reduced (71). The outcome occurred because the activity of proinflammatory cells such as Th1 and Th17 cells were inhibited, but Treg were unaffected. Thus, the representation of the two populations changed with Treg becoming enriched (71). Findings from another group demonstrated the importance of hypoxia associated glycolytic molecules in SK pathogenesis (72). Besides glycolytic metabolism, T effectors, and Treg also show differences in amino acid metabolism. Amino acids, particularly glutamine, plays a key role in fueling effector T cell differentiation, whereas Treg are less dependent on amino acids for their energy (68). In addition, microbial metabolites such as short chain fatty acids or diets rich in vitamin A promote Treg differentiation and function in the gut (73, 74). Additional metabolic differences are also under investigation such as the differential use of lipid oxidation and synthesis pathways. Thus, manipulating metabolic pathways to influence inflammatory lesions is in the early stages of investigation but the approach has great potential and could be more affordable than many of the alternatives. However, the strategy will need considerable scrutiny especially if used for long term therapy. Indeed, our own studies have already documented some untoward consequences when glucose metabolism is compromised during the time when virus is actively replicating.

## Contribution of Corneal Nerve Damage to SK Pathology

Following corneal infection, HSV-1 replicates in the epithelial cells and gains access to the sensory nerve endings which drain the corneal tissues and can travel up (retrograde) to the TG where the virus establishes latency. The virus travels back (anterograde) from the TG to the cornea through the sensory nerves after reactivation. HSV-1 corneal infection can result in destruction of corneal nerve endings resulting in loss of corneal sensitivity (75). Such loss of corneal sensation and nerve function is one of the hall marks of SK in humans and is commonly referred to as neurotrophic keratopathy (76). Evidence from recent studies in mice have shown that sympathetic nerves innervate the cornea and replace the sensory nerve endings lost after HSV-1 corneal infection (75). These sympathetic nerves enhance the infiltration of immune cells resulting in severe corneal inflammation and pathology. A surgical procedure called superior cervical ganglionectomy (SCGx) that removes sympathetic nerves from the cornea helped to alleviate SK severity. Of note, after the SCGx procedure, the sensory nerves reinnervated the cornea resulting in the restoration of corneal sensitivity (75). The exact mechanisms involved in sympathetic corneal innervation are not known and this aspect requires further examination. It is likely that immune cells such as CD4 T cells could play a key role, as their depletion resulted in reversing nerve damage (77). Findings from another study suggest that the molecule involved in cell migration, semaphorin 7A might play a role in the corneal nerves degeneration and regeneration process in HSV-1 infected mice (78). The cytokine IL-6 produced during the inflammatory response to HSV-1 infection in the cornea might also be responsible for causing corneal sensory nerve damage (79).

## Concluding Remarks

Stromal keratitis (SK) caused by HSV-1 corneal infection is a debilitating disease and one of the major causes of vision loss due to an infectious agent. As T cells are the primary orchestrators of SK, steps to improve the host environment which favors Treg over pathogenic Th1/Th17 cells is likely to help ease the severity of SK lesions. In addition, it is becoming increasingly clear from recent developments that metabolism plays a key role in immune function. Thus, as discussed in this review, understanding the events involved in pathogenesis along with key molecules and metabolic pathways involved in inflammation and applying this knowledge to develop better therapies might help control SK in the future.

## Author Contributions

All authors listed have made a substantial, direct and intellectual contribution to the work, and approved it for publication.

### Conflict of Interest Statement

The authors declare that the research was conducted in the absence of any commercial or financial relationships that could be construed as a potential conflict of interest.
